# Temporal changes in the systemic concentrations of retinoids in pregnant and postpartum women

**DOI:** 10.1371/journal.pone.0280424

**Published:** 2023-02-16

**Authors:** Hyunyoung Jeong, Abigail T. Armstrong, Nina Isoherranen, Lindsay Czuba, Amy Yang, Katelynn Zumpf, Jody Ciolino, Elizabeth Torres, Catherine S. Stika, Katherine L. Wisner

**Affiliations:** 1 Department of Industrial and Physical Pharmacy and Pharmacy Practice, College of Pharmacy, Purdue University, West Lafayette, IN, United States of America; 2 Department of Pharmacy Practice, College of Pharmacy, Purdue University, West Lafayette, IN, United States of America; 3 Department of Pharmaceutics, School of Pharmacy, University of Washington, Seattle, WA, United States of America; 4 Department of Preventive Medicine (Biostatistics), Northwestern University Feinberg School of Medicine, Chicago, IL, United States of America; 5 Department of Psychiatry and Behavioral Sciences, Northwestern University Feinberg School of Medicine, Chicago, IL, United States of America; 6 Department of Obstetrics and Gynecology, Northwestern University Feinberg School of Medicine, Chicago, IL, United States of America; Laboratoire de Biologie du Développement de Villefranche-sur-Mer, FRANCE

## Abstract

Retinoids and vitamin A are essential for multiple biological functions, including vision and immune responses, as well as the development of an embryo during pregnancy. Despite its importance, alterations in retinoid homeostasis during normal human pregnancy are incompletely understood. We aimed to characterize the temporal changes in the systemic retinoid concentrations across pregnancy and postpartum period. Monthly blood samples were collected from twenty healthy pregnant women, and plasma concentrations of retinol, all-*trans*-retinoic acid (*at*RA), 13-*cis*-retinoic acid (13*cis*RA), and 4-oxo-retinoic acids were measured using liquid chromatography-tandem mass spectrometry. Significant decreases in 13*cis*RA concentrations over the pregnancy were observed, with rebound increases in retinol and 13*cis*RA levels after delivery. Of note, *at*RA concentrations exhibited a unique temporal pattern with levels peaking at mid-pregnancy. While the 4-oxo-*at*RA concentration was below the limit of quantification, 4-oxo-13*cis*RA was readily detectable, and its temporal change mimicked that of 13*cis*RA. The time profiles of *at*RA and 13*cis*RA remained similar after correction by albumin levels for plasma volume expansion adjustment. Together, the comprehensive profiling of systemic retinoid concentrations over the course of pregnancy provides insights into pregnancy-mediated changes in retinoid disposition to maintain its homeostasis.

## Introduction

**Fig 1:**
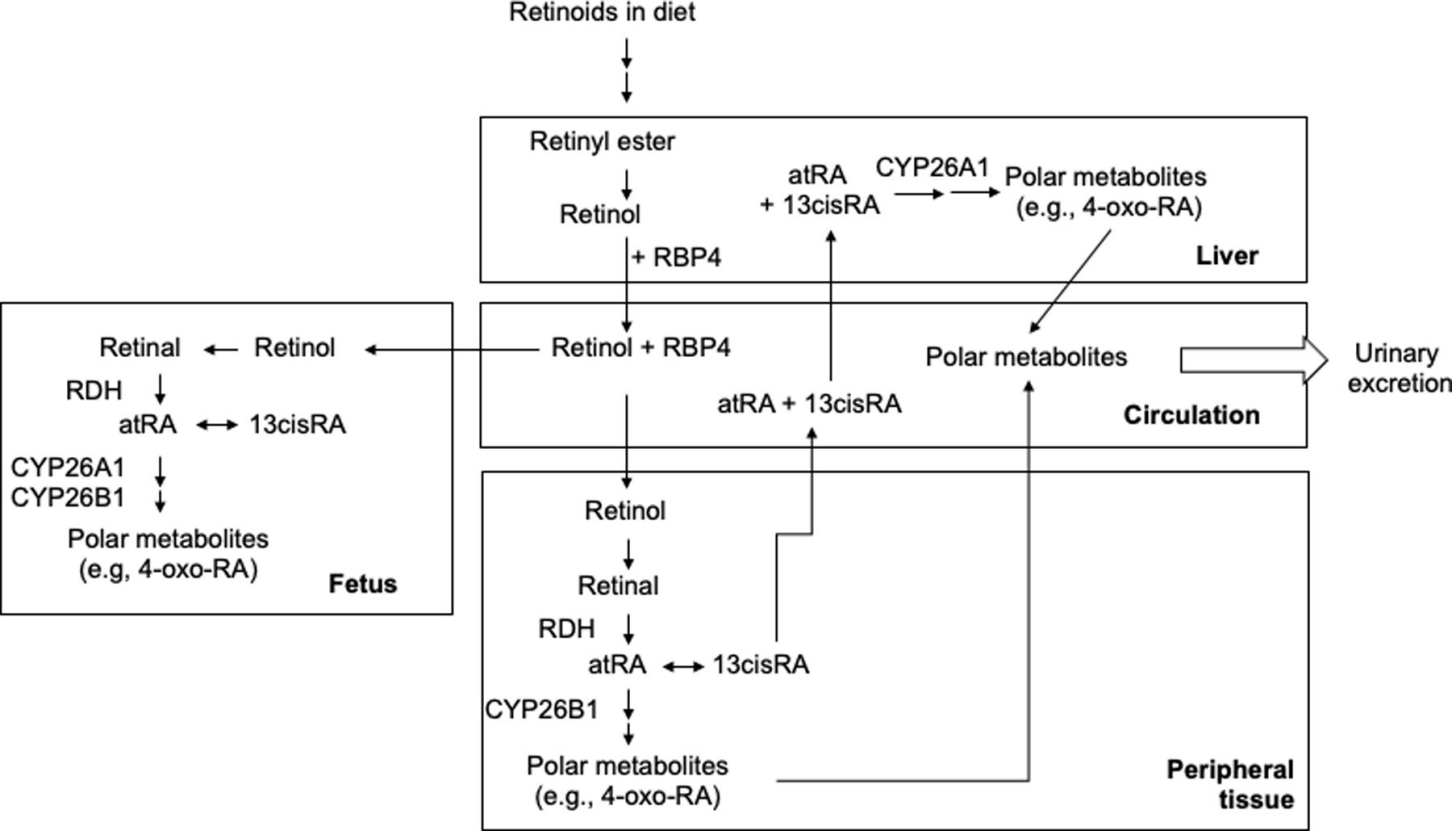
Retinoid disposition during pregnancy. Retinoids are stored in the liver as retinyl esters and mobilized to peripheral tissues as retinol that is subsequently converted to retinal (by retinol dehydrogenases, RDH) and then to bioactive all-*trans*-retinoic acid (*at*RA) and 13-*cis*-retinoic acid (13*cis*RA) by aldehyde dehydrogenases. Retinoic acids are converted to oxidative metabolites by cytochrome P450 enzymes including CYP26A1 (liver and embryo/fetus) and CYP26B1 (extrahepatic tissue).

Vitamin A is an essential nutrient involved in multiple biological processes including vision, reproduction, growth, development, and differentiation [1]. Retinoids (vitamin A and derivatives) are derived from the diet as retinyl esters or β-carotene (Fig 1). In the intestinal cells, retinyl esters or β-carotene are hydrolyzed and re-processed into chylomicrons. After peripheral lipolysis and hepatic processing, retinoids are stored in the hepatic stellate cells as retinyl esters, mainly as retinyl palmitate and retinyl stearate [2]. The stored retinoids are mobilized to peripheral tissues by (hepatic) enzyme-mediated hydrolysis of retinyl esters to retinol. Retinol is subsequently released into systemic circulation as a complex with retinol-binding protein 4 (RBP4). Retinol-RBP4 complexes are taken up by the peripheral tissues where retinol is converted to retinal by retinol dehydrogenases (RDH), followed by retinal oxidation to retinoic acid by retinaldehyde dehydrogenases (RALDHs). Retinoic acid is the bioactive molecule for most retinoid biological functions (except for vision) [3]. The main bioactive isomer all-*trans*-retinoic acid (*at*RA) is detected in the blood of humans and animals while endogenous 13-*cis*-retinoic acid (13*cis*RA) has been mainly detected in human blood and tissues. Retinoic acids undergo oxidative metabolism into 4-oxo-retinoic acids by cytochrome P450s (CYPs), including CYP26A1 (liver, embryo/fetus), CYP26B1 (extrahepatic tissue), CYP2C8 (liver) and CYP3A (liver) [4, 5]. 4-Oxo-*at*RA and 4-oxo-13*cis*RA have been detected previously in human blood although the concentrations of 4-oxo-*at*RA were below the detection limit in most samples [6].

During pregnancy, retinoids are essential for the maintenance of the placenta as well as the development of the embryo [1]. However, current data suggests that as long as sufficient vitamin A is provided to the fetus from maternal stores the embryo/fetus can self-regulate retinoic acid concentrations and gradients necessary for appropriate morphogenesis and fetal development. Retinoic acid controls the expression of key developmental genes and determines the pattern formation of different organs in the embryo [7]. Considering the key roles of retinoids in embryonic development, retinoid concentrations during pregnancy have been extensively studied in rodents and humans. Systemic retinol concentration decreased over the course of human pregnancy, followed by rebound increases after delivery [8–10]. In mice, liver *at*RA concentrations decreased during pregnancy, suggesting altered retinoid homeostasis or signaling during pregnancy.

While the changes in retinol disposition have been studied during gestation in pregnant women, reports on how pregnancy affects the systemic levels of retinoic acids and their oxidative metabolites are scarce [10, 11] and few studies have compared pregnancy retinoid levels to the same individuals post-partum or pre-pregnancy. Distinct temporal changes in the plasma *at*RA and 13*cis*RA concentrations were reported throughout pregnancy and postpartum period, but the study was limited as it involved only one participant [11]. Plasma concentrations of retinoic acids and oxidative metabolites were reported in twenty-three healthy women in mid-late pregnancy, but the data were collected in a limited number of time points with the results in early pregnancy missing [10]. The objective of this study was to explore the temporal changes in maternal systemic concentrations of retinoid species (including retinol, retinoic acid, and its oxidative metabolites), from early gestational time points to postpartum periods.

## Materials and methods

### Participants

The participants were recruited at the Northwestern University. The study was approved by Northwestern IRB and all participants provided written informed consent. Inclusion criteria were: (1) Women aged 18–45 years, (2) English speaking, (3) pregnant, as early as possible and prior to 13 weeks gestation (patients were recruited at their first obstetric appointment early in pregnancy), (4) singleton gestation (for measurement of inter-individual variability), (5) able to present for blood sampling between 8 AM-12 PM, (6) hematocrit ≥28 during pregnancy. Exclusion criteria were: (1) Systemic inflammatory diseases (e.g., rheumatoid arthritis and systemic lupus erythematosus), diabetes, hypertension, chronic liver or renal diseases, hyper- or hypothyroidism, (2) taking supplemental vitamin A other than a prenatal multivitamin, (3) chronic use of prescription drugs that are substrates or inhibitors of CYP2D6 (to avoid drug-drug interactions and potential interference with CYP enzyme activity evaluation), (4) history of pre-eclampsia or gestational diabetes in previous pregnancy, and (5) self-reported pre-pregnancy BMI greater than 30. We planned to enroll 20 subjects and allowed for 20% dropout (targeting N = 16 for analysis). This allowed for detection of a moderate to large change from one timepoint to another of 0.7 to 0.8 standard deviation units with 80% power at the 5% level of significance.

### Blood sample collection

Longitudinal blood collection from pregnant women followed the recommended design features from the FDA PK guidance (i.e., women serving as their own controls across pregnancy and postpartum to evaluate changes associated with the evolving physiology) [12]. Blood samples were collected from each woman at four-week intervals (± 2 weeks) over the course of pregnancy and postpartum period until two months postpartum. To prevent diurnal variations in retinoid levels from confounding our results, all blood samples were collected at the same time of day (e.g., 8 AM to 12 PM) without any restrictions on food intake. Because retinoids are sensitive to white light [13], the blood samples were collected into foil-wrapped tubes with the intensity of light in the room recorded (the maximum light exposure was 42 lux). The plasma samples were stored in amber freezer vials until analysis at the University of Washington. Phlebotomy was performed at the Diagnostic Testing Center (DTC) or The Asher Center at Northwestern University. Vital signs (blood pressure and pulse) were checked prior to phlebotomy. Participants were asked to report the name of prenatal vitamin at every research visit and any significant restrictions or changes in their diet.

### Chemicals

All-*trans*-retinoic acid-d_5_ and 13-*cis*-retinoic acid-d_5_ were purchased from Cayman Chemical (Ann Arbor, MI). Retinol-d_6_ was purchased from Cambridge Isotopes Laboratories (Tewksbury, MA). All-*trans-*retinoic acid, 13-*cis-*retinoic acid, and retinol were purchased from MilliporeSigma (Burlington, MA). 4-Oxo-all-*trans*-retinoic acid, 4-oxo-13-*cis*-retinoic acid, 4-oxo-13-*cis*-retinoic acid-d_3_ was from Santa Cruz Biotechnology (Dallas, TX). Optima LC/MS grade acetonitrile, water, and formic acid were from Thermo Fischer Scientific (Waltham, MA). Human serum (DC Mass Spect Gold MSG 4000) was purchased from Golden West Biologics (Temecula, CA).

### Retinoid measurements

Endogenous retinoids were quantified from human plasma using previously established ultra-high-performance liquid chromatography mass spectrometry (UHPLC-MS/MS) methods [6, 14]. All sample preparation was performed on ice and under yellow-red lights as previously described [10]. In brief, 60 μL of human plasma samples, standard curve samples, and spiked quality control samples were protein precipitated with 120 μL of ice-cold acetonitrile containing 50 nM 4-oxo-13-*cis*-retinoic acid-d_3_, all-*trans-*retinoic acid-d_5_, 13-*cis*-retinoic acid-d_5_, and 750 nM retinol-d_6_. Following protein precipitation, samples were mixed by pipetting, centrifuged twice, and cleared supernatant transferred to a plate for UHPLC-MS/MS analysis. For retinol analysis, 10 μL of cleared supernatant was diluted to 200 μL total volume with acetonitrile before analysis. Plasma retinoids were separated using an Agilent 1290 Infinity II UHPLC (Santa Clara, CA) coupled to an Ascentis Express RP Amide column (2.7 μm; 150 mm × 2.1 mm) and detected on an AB Sciex 5500 (retinol) or 6500 (RA and metabolites) qTrap Q-LIT mass spectrometer (Foster City, CA) operated in positive ion APCI mode. All UHPLC-MS/MS parameters were as previously described [6, 14]. Data analysis was performed using MultiQuant 2.1.1 (Sciex). For each run, at least 2/3 of QC samples at each concentration quantified within 15% of the nominal concentration in accordance with bioanalytical guidelines [15].

### RBP4 measurement

Serum retinol-binding protein 4 (RBP4) was quantified using a Human RBP4 Quantikine ELISA Kit (R&D Systems, Minneapolis, MN) according to manufacturer’s recommendations and as previously described [16].

### Albumin measurement

Plasma albumin levels were measured by bromocresol green albumin Assay (Sigma-Aldrich, St. Louis, MO) using bovine serum albumin standards.

### Statistical analysis

To determine the change in retinol concentrations across pregnancy and postpartum, a segmented regression analysis was performed [17] utilizing a linear mixed effects model, random intercepts for participant to account for repeat measurements, and the following fixed effects: weeks from last menstrual period, an indicator for after postpartum, and number of weeks since delivery. Adjusted analysis plan also included the time of blood draw, pre-pregnancy body weight, weight gain, the intensity of light during sample collection (in consideration of light sensitivity of retinoid compounds), and the amount of vitamin A intake as covariates. Analyses were performed using R (version 4.0.3, 2020, The R Foundation) and SAS and assumed a two-sided 5% level of significance. Given the exploratory nature of the analyses, there were no adjustments made for multiple hypothesis tests.

## Results

Twenty participants enrolled in the study. Three participants (15% of the participants) were lost to follow-up (the last visits for these participants were 20–24, 32–36, and 36+ weeks of gestation). The data obtained prior to their loss were included in the analysis. Seventeen participants completed the study protocol and contributed monthly blood samples during pregnancy and two additional samples postpartum, one month apart. The participant characteristics are shown in Table 1. The average age of participants was 33 years at ~12 weeks of gestation. The mean collection times for the first and the second postpartum samples were 3.9 and 8.2 months postpartum (ranging 2.3–5.6 and 6.0–11.3 months, respectively). Plasma concentrations of retinoids (retinol, *at*RA, 13*cis*RA, and 4-oxo-RA), RBP4, and albumin [as a marker of plasma volume expansion [18]] were measured in a total of 156 samples. Changes in their concentrations during pregnancy and postpartum period were analyzed after pre-specified adjustments for the time of blood draw, pre-pregnancy weight, changes in body weight, the intensity of light during sample collection, and the amount of vitamin A intake (via prenatal vitamins). None of the covariates exhibited a significant association with any of the retinoids measured in this study.

**Table 1:** Participant characteristics.

	Total (N = 20)
**Age**	
Mean (SD)	33.3 (4.0)
[Min, Max]	[25.0, 43.0]
**Weeks of Gestation**	
Mean (SD)	11.7 (1.3)
[Min, Max]	[9.3, 15.0]
**Race**	
Asian	2 (10.0%)
Black or African American	2 (10.0%)
White or Caucasian	15 (75.0%)
Declined to Answer	1 (5.0%)
**Hispanic or Latino**	
No	18 (90.0%)
Yes	2 (10.0%)
**Amount of daily Vitamin A supplementation (IU)**	
Mean (SD)	3540 (934)
[Min, Max]	[2000, 5200]
Missing	1 (5.0%)
**Pre-pregnancy BMI**	
Mean (SD)	23.2 (2.9)
[Min, Max]	[19.0, 29.7]
**Pre-pregnancy weight (lbs)**	
Mean (SD)	138 (20)
[Min, Max]	[114, 185]

### Retinol

**Fig 2:**
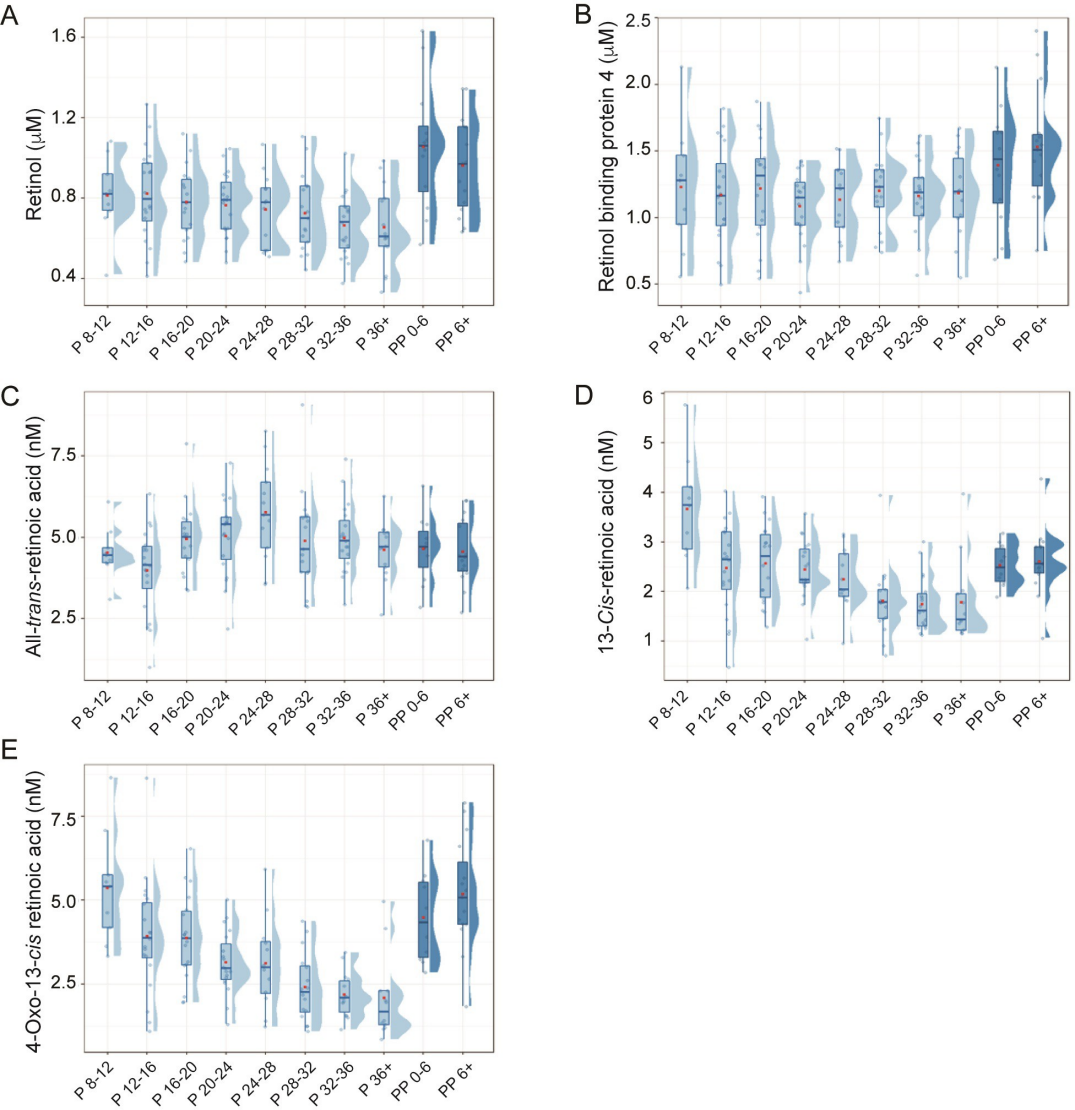
Longitudinal changes in retinol (A), RBP4 (B), *at*RA (C), 13*cis*RA (D), and 4-oxo-13*cis*RA (E) during pregnancy and postpartum periods. The plots depict the mean (red dots), median (horizontal bars), 25^th^ and 75^th^ quantiles (upper and lower box ranges, respectively), and 1.5 interquartile ranges. The numbers after P and PP indicate the weeks of gestation and the number of weeks passed after delivery, respectively.

Retinol concentrations ranged from 0.33 to 1.63 μM with the average of 0.79±0.23 μM. Retinol concentration exhibited a decreasing trend across pregnancy although the changes were marginally significant [β: -0.01, 95% confidence interval (CI) -0.01–0.00; *p*-value = 0.0535] (Fig 2A). On average, the retinol concentration dropped by 20% from the first to last sampling point during pregnancy (i.e., 0.81 to 0.65 μM). Retinol concentrations significantly increased after delivery. The average increase in retinol concentration shortly after delivery was 0.50 μM (95% CI 0.33–0.67; *p*<0.0001). During the later postpartum period, retinol levels decreased to a small extent (by 0.03 μM per week; CI 0.01–0.05) but in a statistically significant manner (*p* = 0.0085).

Of note, a significant number of plasma samples had retinol concentrations below 1.05 or 0.7 μM, the clinically defined retinol concentrations for vitamin A insufficiency and deficiency, respectively [10, 19, 20] (Table 2) in nonpregnant women and men.

**Table 2:** Number of participants with retinol plasma concentrations in the vitamin A deficiency (<0.7μM) or insufficiency (0.7–1.05 μM) category.

Gestational Age	Retinol < 0.7 μM (N = 59)	Retinol 0.7–1.05 μM (N = 76)	Retinol > 1.05 μM (N = 21)
P 8–12	2 (22%)	6 (67%)	1 (11%)
P 12–16	6 (30%)	11 (55%)	3 (15%)
P 16–20	6 (33%)	11 (61%)	1 (6%)
P 20–24	7 (37%)	11 (58%)	1 (5%)
P 24–28	5 (38%)	7 (54%)	1 (8%)
P 28–32	9 (53%)	6 (35%)	2 (12%)
P 32–36	10 (53%)	9 (47%)	0 (0%)
P 36+	9 (64%)	5 (36%)	0 (0%)
PP 0–6	2 (17%)	4 (33%)	6 (50%)
PP 6+	3 (20%)	6 (40%)	6 (40%)

The numbers after P and PP indicate the weeks of gestation and the number of weeks passed after delivery, respectively.

### RBP4

RBP4 concentrations ranged from 0.44 to 2.40 μM with an average of 1.22±0.36 μM. The plasma concentrations of RBP4 remained constant over the course of pregnancy and exhibited an increasing trend of ~10% after delivery (Fig 2B).

### *at*RA and 4-oxo-*at*RA

**Fig 3:**
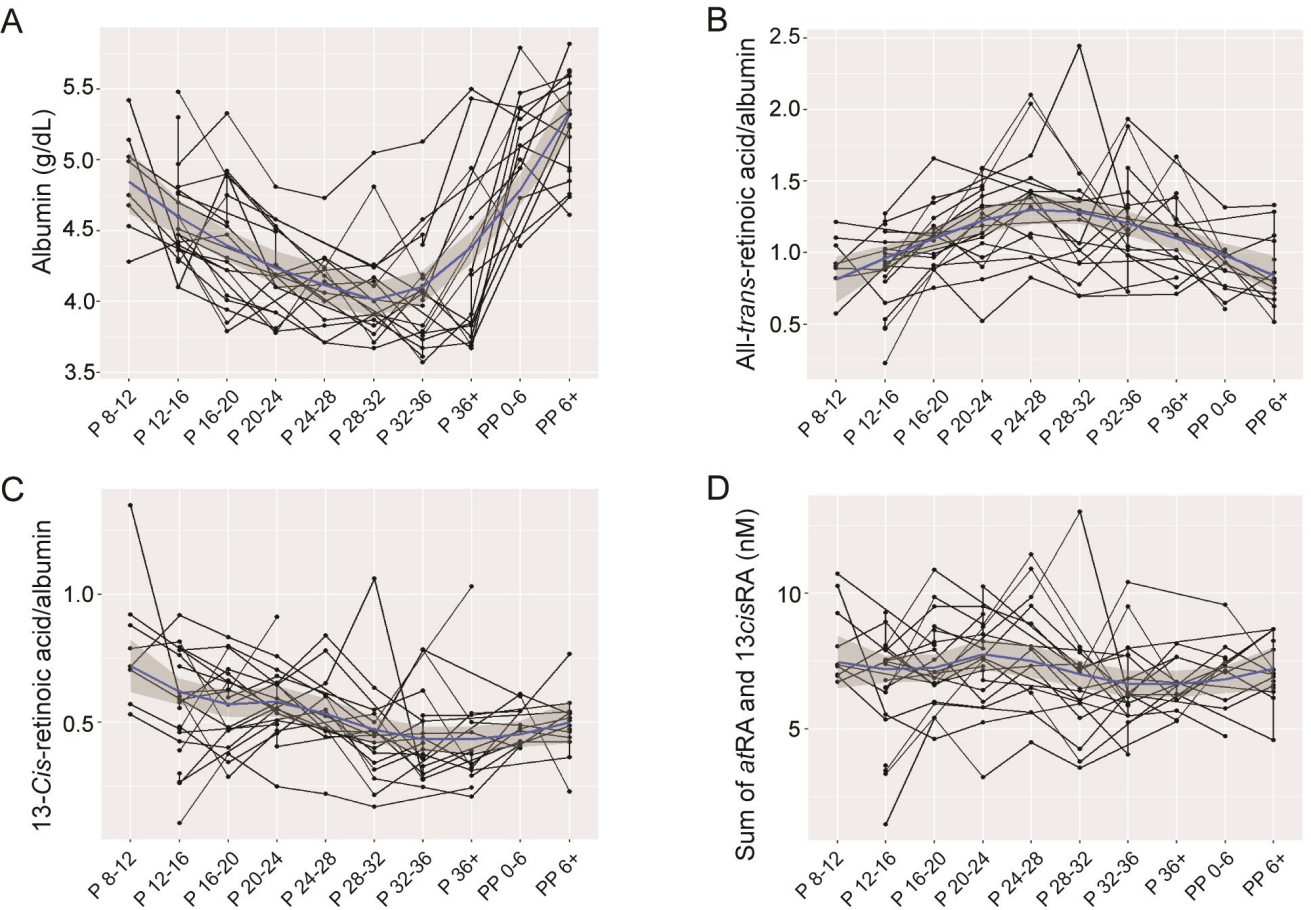
Longitudinal changes in the concentrations of albumin (A), albumin-corrected *at*RA (B), albumin-corrected 13*cis*RA (C), and the sum of *at*RA and 13*cis*RA (D) during pregnancy and postpartum periods. The blue line is from LOWESS (locally weighted scatterplot smoothing) regression, and gray area represents the 95% uncertainty around the smoothed line estimate. P and PP denote pregnancy and postpartum periods, respectively. The numbers after P and PP indicate the weeks of gestation and the number of weeks passed after delivery, respectively.

*at*RA concentrations ranged from 1.01 to 9.07 nM with an average of 4.78±1.23 nM. 4-Oxo-*at*RA levels were below limit of quantification (i.e., 1 nM), which is consistent with the previous report in nonpregnant subjects [6]. The changes in *at*RA levels in either pregnancy or postpartum periods (Fig 2C) did not fit a linear model (*p*>0.05). Of note, *at*RA concentrations showed an increasing trend until mid-pregnancy, followed by a decrease through the late pregnancy and postpartum period. The highest average *at*RA concentration was obtained at weeks 24–28 (5.77±1.48 nM), the level 1.3-fold higher than that at week 8–12 (4.52±0.80 nM). The rise in *at*RA concentration at mid-pregnancy became more prominent when *at*RA concentrations were corrected by albumin for plasma volume expansion adjustment (Fig 3B). The corrected *at*RA levels were on average 1-8-fold higher at mid-pregnancy as compared to the early gestational time point.

### 13*cis*RA and 4-oxo-13*cis*RA

13*cis*RA concentrations ranged 0.47–5.77 nM with the average of 2.32±0.86 nM. The concentrations of 4-oxo-13*cis*RA were comparable, with the average of 3.52±1.66 nM (ranging 0.86–8.65 nM). Plasma concentrations of 13*cis*RA significantly decreased during pregnancy by 0.04 nM per week (95% CI 0.02–0.06, *p* = 0.0013) (Fig 2D). Similar changes were observed when 13*cis*RA concentrations were corrected by albumin (Fig 3C). After delivery, the concentration increased by an average of 0.90 nM (95% CI 0.07–1.72, *p* = 0.034). Insignificant changes in 13*cis*RA were observed across the postpartum period. Throughout the pregnancy and postpartum period, the sum of *at*RA and 13*cis*RA (two bioactive RA species) remained constant (Fig 3D). In contrast to 4-oxo-*at*RA, which was undetectable, 4-oxo-13*cis*RA was readily detected in all samples, likely due to the slow elimination of 4-oxo-13*cis*RA (half-life >20 h) [21]. The plasma concentration of 4-oxo-13*cis*RA exhibited a similar pattern to that of 13*cis*RA (Fig 2E). 4-Oxo-13*cis*RA concentrations significantly decreased during pregnancy by 0.08 nM per week (95% CI 0.04–0.12, *p* = 0.0001). After delivery, 4-oxo-13*cis*RA level increased by 2.59 nM on average (95% CI 1.21–3.97, *p* = 0.0003).

## Discussion

This is the first comprehensive report of longitudinal monitoring of retinoid levels analyzed in the same subjects of 17 pregnant women over the course of pregnancy, encompassing early pregnancy to postpartum periods. The concentrations of retinol, retinoic acids, and oxidative metabolites were measured in monthly collected blood samples, followed by adjustment for multiple variables. Considering that food intake or subchronic vitamin A supplementation (27.6 mg/day retinyl palmitate; equivalent to ~17 times the recommended daily allowance for adult men) does not affect plasma retinoid concentration significantly [22, 23], detailed nutritional analysis of food for vitamin A content was not performed in this study.

We found that retinol concentrations decreased over the course of pregnancy, followed by a 1.6-fold increase after delivery (compared to the last sampling point before delivery). While previous studies have reported similar decreases in retinol concentrations from early to late gestational time points [8, 24], this is the first where the extent of changes was examined in the same subjects. The decrease in plasma retinol concentrations has been attributed in part to vascular volume expansion during pregnancy, which typically resolves within two weeks after delivery [25]. Indeed, the plasma albumin level [used as a marker for pregnancy plasma expansion [18]] decreased by 13% on average in our participants from early pregnancy to the last sampling points before delivery (from 4.9 to 4.2 g/dL). In comparison, the plasma retinol levels decreased by 19% (from 0.81 to 0.65 μM) on average during the same period, similar to the extent reported previously [8, 24]. The additional drop in maternal plasma retinol concentration (as compared to the decrease in albumin level) could reflect altered retinoid homeostasis during pregnancy. In mice, significant decreases in maternal plasma retinol levels and hepatic retinyl ester contents (by 80% and 40%, respectively) as compared to the pre-pregnancy levels were reported [26]. This was accompanied by concurrent increases in embryonic retinol contents (per gram tissue), suggesting the transfer of maternal hepatic retinoid stores to the embryo. Although to a smaller extent, the decrease in maternal plasma retinol levels may reflect the additional route of maternal retinol clearance by embryo and fetus.

The retinol concentrations observed in this study, ranging 0.3–1.6 μM (average 0.79 μM), are lower than those reported in healthy Norwegian and Swedish pregnant women (ranging 1.4–1.8 μM) [11, 24], comparable to results from pregnant women in the US urban population (0.2–1.9 μM) [10, 20], and higher than those in pregnant women or nonpregnant controls in China (0.3–0.8 μM) [27]. Based on the clinically defined retinol concentrations for vitamin A deficiency, i.e., 0.7 μM [19], our results indicate that 22% and 64% of the study participants were vitamin A deficient in early and late pregnancy (8–12 and >36 weeks of gestation), respectively. However, it is unclear whether the same plasma retinol concentrations should be used to assess dietary sufficiency in pregnant women as in nonpregnant women and men. Vitamin A deficiency in humans takes months to develop; hence, it is unlikely that the relatively short pregnancy timeline is sufficient to deplete liver retinyl ester stores to the level of deficiency with a prompt return to sufficiency after delivery without intervention. Rather, the lower retinol concentrations observed in pregnancy are likely a result of physiological processes that occur during pregnancy that result in a different relationship between maternal liver retinoid stores and plasma concentrations than observed in nonpregnant women and men. Our study was uniquely designed to consider the dynamics of maternal circulating retinoid concentrations in this context as we compared the time course of changes during pregnancy to those postpartum. The high prevalence of low plasma retinol concentrations in our study population appears in line with previous reports that 62% and 48% of women at term pregnancy in Bronx inner city and Seattle, respectively, had plasma retinol concentrations lower than 1.05 μM) [10, 20]. The prevalence of vitamin A insufficiency in US female population (aged 17 to 42 years) is much lower (i.e., less than 10%) than that in pregnant women [28], indicating that pregnancy poses a risk for further decreases in plasma retinol concentrations. Of note, except in geographical areas with a severe public health problem related to vitamin A deficiency, the WHO does not recommend vitamin A supplementation during pregnancy [29].

RBP4 is responsible for mobilization of retinol from the liver to extrahepatic tissues. RBP4 plasma levels generally correlate with retinol concentrations. For example, upon retinol depletion, RBP secretion into the circulation as retinol-RBP complex decreases, leading to lower plasma RBP levels [30]. In this study, despite the gradual decreases in retinol concentration during pregnancy, RBP4 concentration did not change. A tendency of a moderate (~20%) increase in RBP4 level after delivery was noted, likely reflecting the return to the nonpregnant, normal distribution volume.

Most biological actions of retinoids are mediated by retinoic acid, including *at*RA and 13*cis*RA. Interconversion among these isomers has been reported in both *in vitro* and *in vivo* systems [31] and is mediated by both nonenzymatic and enzymatic reactions [32–35]. We found that the sum of 13*cis*RA and *at*RA maternal concentrations remained constant throughout the pregnancy and postpartum periods, but their temporal changes were distinct. *at*RA is the most bioactive retinoic acid that binds to intracellular binding proteins for transcellular transport to the nucleus for gene regulation [36] and enzymes for oxidative metabolism [37, 38]. *at*RA concentrations exhibited a unique temporal profile that they rose at mid-pregnancy. A similar finding (i.e., increased *at*RA levels at mid-term pregnancy) was reported in a study of one pregnant woman; *at*RA concentrations increased in the second trimester (by ~40% as compared to early gestational time point) [11]. This indicates that specific metabolic processes in the mom result either in increased formation of *at*RA or decreased clearance of *at*RA at mid-gestation. On the other hand, 13*cis*RA concentrations decreased over time during pregnancy and then increased (by 0.7 nM on average) after delivery, suggesting 13*cis*RA clearance may be increased during pregnancy. The temporal changes in 4-oxo-13*cis*RA levels mimicked that of 13*cis*RA [23].

The finding that 13*cis*RA and *at*RA concentrations exhibit distinct temporal changes over the course of pregnancy is intriguing, considering widespread interconversion between *at*RA and 13*cis*RA in tissues. Isomerization between *at*RA and 13*cis*RA has been reported by rat intestinal epithelium [31], human cell line (i.e., HepG2), and primary human hepatocytes [39] as well as rat conceptual homogenates [40]. The differential temporal patterns of *at*RA and 13*cis*RA concentrations during pregnancy may be attributed to differences in the elimination pathways of *at*RA and 13*cis*RA. *at*RA elimination is much faster than 13*cis*RA; terminal half-lives were ~0.5 and >10 h for *at*RA and 13*cis*RA, respectively, after a very low intravenous dose (0.0125 mg/kg) in monkeys [41]. The major elimination pathway of *at*RA is oxidative metabolism by CYP26 isoforms that are expressed in multiple tissues including maternal liver, placenta, and fetus [4, 42]. *at*RA metabolism by CYP26 enzymes is modulated by *at*RA binding to the intracellular RA-binding proteins that channel *at*RA to CYP26 after binding with high affinity [37, 43]. Hence the increased *at*RA concentrations may imply lower expression of CYP26 during pregnancy or increased expression of intracellular RA-binding proteins limiting *at*RA metabolism. Hepatic CYP26 expression increased (rather than decreased) at term pregnancy in mice [44], potentially reflecting *CYP26* promoter activation by *at*RA [45], although it is unknown whether corresponding changes in CYP26 expression occur in human pregnancy and in extrahepatic tissues that may be the major *at*RA clearing organs. How pregnancy alters the expression of intracellular RA-binding proteins is entirely unknown.

The limitations of this study include the lack of diversity in the participant population, small sample size (due to minimal information to guide sample size and power calculations and parameter behavior), the lack of retinoid analysis in tissues other than maternal blood (such as maternal liver, placenta, and fetus) to fully characterize the changes in retinoid homeostasis during human pregnancy. Despite the limitations, the individual and comprehensive profiling of systemic retinoid concentrations over the course of pregnancy and the postpartum period has revealed distinct time profiles of different retinoid species and provided insights into how retinoid homeostasis is maintained during pregnancy with implications to understanding maternal vitamin A homeostasis and dietary guidance for vitamin A supplementation.

## Supporting information

S1 Dataset (XLSX)
