# Correction: Temporal changes in the systemic concentrations of retinoids in pregnant and postpartum women

**DOI:** 10.1371/journal.pone.0314399

**Published:** 2024-11-20

**Authors:** Hyunyoung Jeong, Lindsay Czuba, Nina Isoherranen, Abigail T. Armstrong, Amy Yang, Katelynn Zumpf, Jody Ciolino, Elizabeth Torres, Catherine S. Stika, Katherine L. Wisner

The authors are listed out of order. Please view the correct author order, affiliations, and citation here:

Hyunyoung Jeong^1,2^, Lindsay Czuba^3^, Nina Isoherranen^3^, Abigail T. Armstrong^1^, Amy Yang^4^, Katelynn Zumpf^4^, Jody Ciolino^4^, Elizabeth Torres^5^, Catherine S. Stika^6^, Katherine L. Wisner^5,6^

**1** Department of Industrial and Physical Pharmacy and Pharmacy Practice, College of Pharmacy, Purdue University, West Lafayette, IN, United States of America, **2** Department of Pharmacy Practice, College of Pharmacy, Purdue University, West Lafayette, IN, United States of America, **3** Department of Pharmaceutics, School of Pharmacy, University of Washington, Seattle, WA, United States of America, **4** Department of Preventive Medicine (Biostatistics), Northwestern University Feinberg School of Medicine, Chicago, IL, United States of America, **5** Department of Psychiatry and Behavioral Sciences, Northwestern University Feinberg School of Medicine, Chicago, IL, United States of America, **6** Department of Obstetrics and Gynecology, Northwestern University Feinberg School of Medicine, Chicago, IL, United States of America.

Jeong H, Czuba L, Isoherranen N, Armstrong AT, Yang A, Zumpf K, et al. (2023) Temporal changes in the systemic concentrations of retinoids in pregnant and postpartum women. PLOS ONE 18(2): e0280424. https://doi.org/10.1371/journal.pone.0280424.
